# Mucoid degeneration of the anterior cruciate Ligament: a case report

**DOI:** 10.11604/pamj.2013.15.59.2534

**Published:** 2013-06-20

**Authors:** Khalid Ibn el Kadi, Florian Marcaillou, Stephane Blanc, Bassam Salloum, Cyril Dimontagliari, Fawzi Boutayeb

**Affiliations:** 1 Department of Orthopedic Surgery (A), UH Hassan II,Fes, Morocco; 2 Department of Orthopedic Surgery, Rene Dubos Hospital of Pontoise, Paris, France

**Keywords:** Anterior cruciate ligament, arthroscopy, Magnetic Resonance Imaging, mucoid degeneration

## Abstract

We report a case of mucoid degeneration of the anterior cruciate ligament (ACL). Mucoid degeneration of the ACL is a very rare cause of knee pain. There have been only some reported cases of mucoid degeneration of the ACL in the English literature. We reviewed previous reports and summarized clinical features and symptoms, including those found in our case. Magnetic Resonance Imaging is the most useful tool for differentiating mucoid degeneration of the ACL from an intraligamentous ganglion or other lesions in the knee joint. If this disease is considered preoperatively, it can be diagnosed easily based on characteristic findings.

## Introduction

The first description of cysts of the anterior cruciate ligament (ACL) in 1924 and was made by Caan [1] from dissection of its work. However, this disease is recent and unknown. This lesion is rare, with a prevalence of between 0.2 and 1.2% [2, 3]. There are few observations in the literature and series of patients are low numbers (five cysts for Kim et al [3] and Huang et al [4], eight Sumen et al [5], 11 King et al [6] and 23 Courroy et al [7] or Boueri et al [8]).

MRI identified two forms of cyst of the ACL. The first is the cyst fluid. This is the cystic form proper, well described in the literature[3, 4, 9]. The second is the cyst infiltrating or mucoid degeneration of the ACL cystic less known [7, 10, 11] found in our study.

## Patient and observation

**Figure 1:**
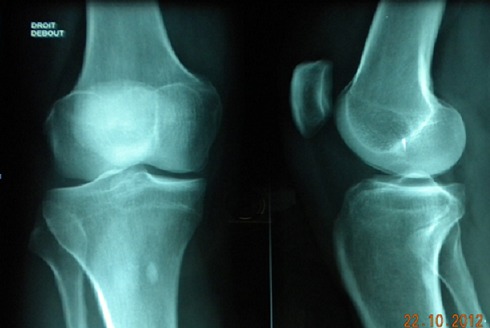
Anteroposterior and lateral radiographs showed a slight degenerative change in the medial side of the knee

A 45 year old woman who had no prior significant trauma was seen at our hospital for evaluation of left knee pain and flexion difficulty. Upon examination, there was no swelling, ballottement of patella or instability. The range of motion of the knee was limited from 0° to 120° with terminal flexion pain. There was a point of tenderness at the medial joint line of the knee. A plain X-ray showed a slight degenerative change in the medial side of the knee (Figure 1).

**Figure 2:**
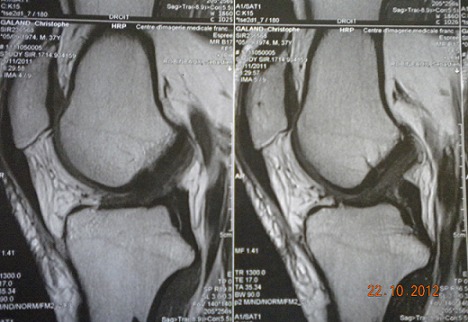
Sagittal T1-weighted image showing inhomogenous high signal intensity of the intact ACL.

**Figure 3:**
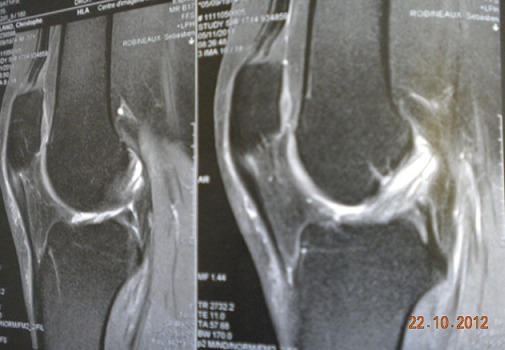
Sagittal T2-weighted image showing increased intraligamentous signal intensity of the ACL.

**Figure 4:**
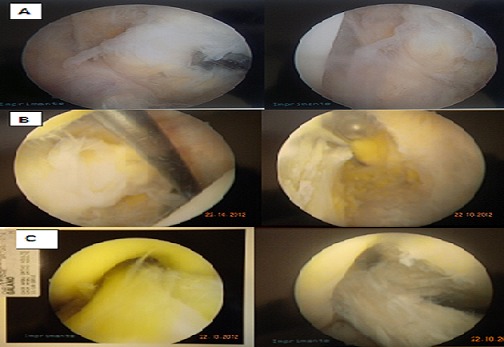
Arthroscopic view of the anterior cruciate ligament (ACL) over the right knee. (A and B) Yellowish discoloration and sclerotic lesions on the lateral portion of the ligament; (C) ACL after debridement

**Figure 5:**
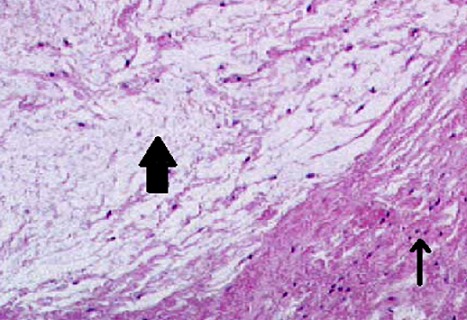
(Histological biopsy specimen from the ACL showing mucoid degenerative tissue (big arrow) and normal ligamentous collagen tissue (small arrow) (H&E, original magnification ×40)

MRI of the right knee showed an increased intraligamentous signal for the ACL with high inhomogeneous intensity and cracking of the external meniscus undisplaced (Figure 2, Figure 3). Based on the patient's history and the MRI findings, we suspected intraligamentous cyst of the ACL and performed arthroscopy. Upon arthroscopy, the ACL was found to be hypertrophied and it was noteworthy that the lateral portion of the ACL was bulbous. Using a longitudinal incision of the ACL, we searched unsuccessfully for mucous discharge from the ligament. We found some yellow and sclerotic lesions on the lateral portion of the ligament and excised these lesions as precisely as possible (Figure 4). Histological examination revealed fragments of mucoid degeneration of the ligament (Figure 5). As of the 20 month follow up, the patient had gained full range of knee motion without pain or instability.

## Discussion

Mucoid degeneration of the ACL is a very rare cause of knee pain. This lesion of the ACL was first reported by Kumar et al [12] in 1999. Since their report, there have been only some cases of mucoid degeneration of the ACL reported in the English literature [13]. In each of these reports, a mass-like structure displaced either the ACL or the PCL. In many of these case reports, the presenting complaint was knee pain with a mechanical block to extension without evidence of knee instability. Kumar et al [12] reported mucoid cystic degeneration of the ACL with gross disruption of the normal appearance of the ligament. They implicated mucoid cystic degeneration of the ACL as a potential cause of knee pain.

This case shows the presence of intraligamentous mucoid degeneration in the ACL as a source of knee pain without instability. To assist in diagnosing the source of atypical knee pain, we recommend the use of MRI in conjunction with diagnostic arthroscopy. Partial ACL debridement does not preclude adequate knee stability. The chief complaint of knee pain correlated with the intraligamentous nature of mucoid degeneration in the ACL and may suggest involvement of the small nerve fibers found in the ACL. After resection of the cyst arthroscopy has no laxity was found and Robert [14] had studied the functional quality of LCA degenerative in preoperative and shown by KT 1000TM these knees were stable. It is also mentioned by McIntyre et al [15] reported a case of atraumatic rupture of the ACL to one year postoperatively after partial resection.

In summary, MRI yields preoperative information that is useful in the diagnosis of and surgical treatment planning for mucoid degeneration of the ACL. If we consider this disease preoperatively, it can be easily diagnosed based on these characteristic findings. Partial excision of the yellow, sclerotic lesions of the ACL results in immediate pain relief and improves the range of movement without any symptom of instability.

## Conclusion

Arthroscopic resection of symptomatic degenerative ACL gives good results but leads to a subjective postoperative progressive laxity in some cases. The prognosis depends on the age and associated injuries. The diagnosis of mucoid degeneration of the ACL should be suspected posterior unusual pain with limitation of flexion. MRI and arthroscopy confirmed the diagnosis.
